# FlexiBAC: a versatile, open-source baculovirus vector system for protein expression, secretion, and proteolytic processing

**DOI:** 10.1186/s12896-019-0512-z

**Published:** 2019-03-29

**Authors:** Régis P. Lemaitre, Aliona Bogdanova, Barbara Borgonovo, Jeffrey B. Woodruff, David N. Drechsel

**Affiliations:** 1 Max Planck Institute of Molecular Cell Biology and Genetics, Pfotenhauerstrasse 108, 01307 Dresden, Germany; 2 Department of Cell Biology, Dept. of Biophysics, UT Southwestern Medical Center, Dallas, TX 75390 USA; 3 Research Institute of Molecular Pathology (IMP), Campus-Vienna-Biocenter 1, 1030 Vienna, Austria

**Keywords:** Baculovirus, Intrinsically disordered proteins, Protein expression, TGF-β

## Abstract

**Background:**

Baculovirus-mediated expression in insect cells is a powerful approach for protein production. However, many existing methods are time-consuming, offer limited options for protein tagging, and are unsuitable for secreted proteins requiring proteolytic maturation, such as TGF-β family growth factors.

**Results:**

To overcome the limitations of traditional baculovirus expression systems, we engineered “FlexiBAC”. This system allows recombinant baculovirus formation inside insect cells and reduces the time between initial cloning and protein production to 13 days. FlexiBAC includes 143 shuttle vectors that append combinations of purification tags, fluorescent markers, proteolytic cleavage sites, trafficking signals, and chemical conjugation tags to the termini of the target protein. This system also overexpresses recombinant furin convertase to allow efficient proteolytic processing of secreted proteins. We demonstrate that FlexiBAC can be used to produce high levels of mature, active forms of TGF-β family growth factors, such as Activin A, as well as other proteins that are typically difficult to reconstitute, such as proteins rich in coiled-coil, low complexity, and disordered domains.

**Conclusions:**

FlexiBAC is a protein expression system for production of both cytosolic proteins and secreted proteins that require proteolytic maturation. The design of FlexiBAC and its expansive complementary shuttle vector system reduces cloning steps and simplifies baculovirus production.

**Electronic supplementary material:**

The online version of this article (10.1186/s12896-019-0512-z) contains supplementary material, which is available to authorized users.

## Background

Protein production using baculovirus-infected insect cells combines biological safety with high-level expression of functional proteins. Eukaryotic proteins produced recombinantly in this system generally adopt a native, folded conformation and contain the appropriate post-translational modifications. The large baculovirus genome (ranging between 80 and 180 kbp, depending on the species) can accommodate large insertions, making it ideal for expressing large heteromeric protein complexes. Moreover, insect cells can grow in serum-free media, which greatly facilitates purification of secreted proteins from the conditioned media.

Despite these advantages, recombinant protein production using baculovirus is time-consuming. The time between initial cloning and protein expression lasted 3 to 4 weeks in the earliest baculovirus expression vector systems that relied on plaque selection of recombinants into the polyhedron locus after homologous recombination in insect cells [1, 2]. To make baculovirus production simpler and faster, more recent protocols have facilitated production of recombinant baculovirus genomes using site-specific transposition in *E. coli* (Bac-to-Bac®, Thermo Fisher Scientific) or homologous recombination in insect cells (flashBAC™, Oxford Expression Technologies). Both systems employ the polyhedrin promoter to drive high-level, late expression of the target protein(s) of interest. While the former system is more popular, the latter system is faster, as it reduces the window between initial cloning and protein expression to ~ 14 days. One major drawback of these commercial systems is that they only offer limited options for tagging the protein of interest: 6xHis, GST, and S-tag options are available, but fluorophores or tags for conjugating chemical probes are not. Purification of difficult proteins would be greatly facilitated by a baculovirus vector system that offers a larger combination of purification and solubility tags. Furthermore, as reconstituted systems are analyzed more and more via light microscopy, there is a pressing need for more fluorophore tagging options.

Most baculovirus expression systems are also not suited for high-level secretion of recombinant proteins. The secretory pathway in insect cells has limited capacity and cannot handle the efflux of recombinant proteins during baculovirus infection [3]. During late infection, baculovirus produce cathepsin and chitinase, two highly-expressed enzymes which are secreted by insect cells. In the wild, these enzymes are essential for liquefaction of the larval host, but they play no role in baculovirus replication in cultured cells. It was proposed that eliminating these proteins would reduce the viral protein load passing through the secretory pathway and would free up room for recombinant target proteins. Indeed, deletion of the genes encoding cathepsin and chitinase dramatically improved secretion of recombinant target proteins; unexpectedly, these mutations also reduced degradation of both secreted and non-secreted target proteins [3, 4]. Thus, knocking out genes encoding cathepsin and chitinase should generally improve the efficiency and versatility of baculovirus expression systems.

Another limitation of baculoviral expression systems is that they are not equipped to handle high-volume post-translational processing of secreted proteins. Many secreted proteins require proteolytic trimming to reach a fully active, mature form. One prominent example is the transforming growth factor beta (TGF-β) member Activin A, which must be cleaved by furin convertases in the Golgi apparatus to become an active signaling ligand [5]. Baculovirus-mediated expression of Activin A largely resulted in the secretion of an inactive pro-form peptide [6]. Co-infection of baculoviruses expressing furin and Activin A greatly increased secretion of mature Activin A, indicating that furin activity is limited due to insufficient endogenous expression levels [7]. Engineering a single baculovirus to express furin in addition to the target gene of interest would increase yields of active TGF-β and other secreted peptides that require furin processing, obviating the need for co-infection.

To overcome these limitations, we designed an open-source baculovirus expression system called FlexiBAC. Our system limits the time between initial cloning and protein isolation to 13 days and incorporates rescue of a doubly defective bacmid to ensure that all infective viruses contain the recombinant target gene. FlexiBAC includes a compatible, modular shuttle vector set (143 vectors) that appends combinations of various purification tags, fluorescent markers, proteolytic cleavage sites, trafficking signals, and chemical conjugation tags to the user’s target protein. To allow high yields of secreted proteins, the FlexiBAC viral genome includes deletions of genes encoding cathepsin and chitinase. We also present a specialized version of FlexiBAC that improves the maturation of secreted proteins requiring furin proteolysis. We demonstrate that FlexiBAC is suited for the expression of proteins deemed difficult to reconstitute, including mature TGF-β family growth factors, intrinsically disordered proteins, as well as proteins containing numerous coiled-coil domains.

## Results

### Overview of the FlexiBAC system

**Fig. 1:**
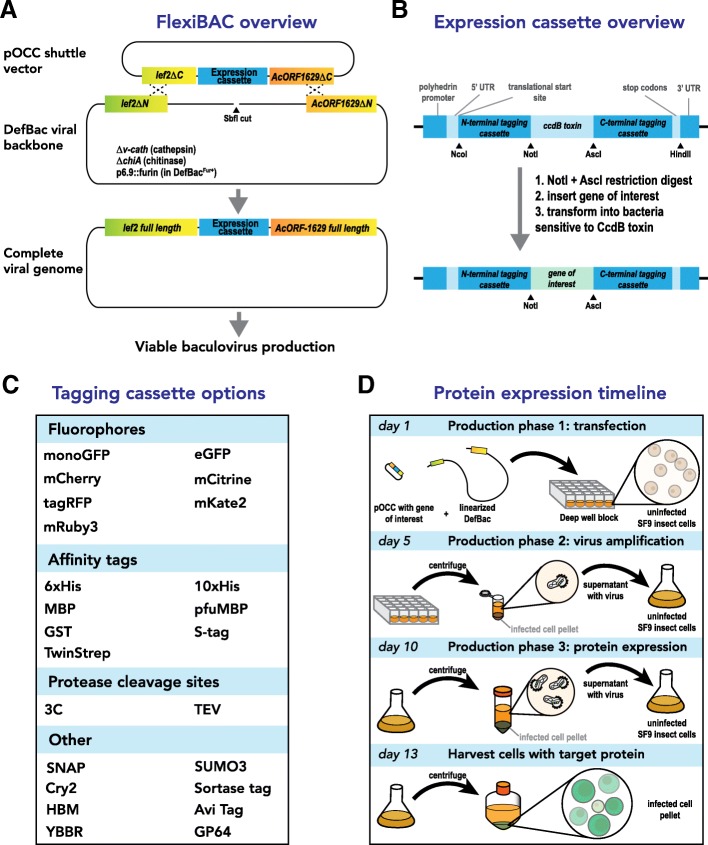
Overview of the FlexiBAC baculovirus expression system. **a** Recombination between a SbfI-linearized defective viral backbone (“DefBac”) and a shuttle vector (“pOCC”, which contains the target gene of interest) creates a viral genome capable of producing infectious virus. Recombination occurs between complementary *lef2* and *AcORF1629* truncations located on the pOCC vector and the DefBac viral backbone. Recombination generates full-length versions of *lef2* and *AcORF1629* genes, which are needed for baculovirus production. No virus is produced without proper recombination, thus eliminating the need for post-production screening for recombinant virus. The DefBac viral backbone also includes deletions in the genes encoding cathepsin and chitinase. A second version of DefBac, called DefBac^Fur+^, expresses the convertase furin. **b** Each pOCC shuttle vector contains a modular expression cassette that allows insertion and swapping of the gene of interest, N-terminal tags, and C-terminal tags using classic cloning techniques (restriction sites are shown with arrowheads). A gene encoding the ccdB toxin selects against plasmids lacking the gene of interest. **c** pOCC shuttle plasmids encode a variety of tags that can be appended to the target protein of interest. Tags are easily combined and swapped to create customizable N-terminal or C-terminal fusions. Descriptions of each tag and the available combinations (143) are shown in Table 1 (see Additional file 1) and available upon request. The most commonly used plasmids (52) are shown in Table 2 (see Additional file 2) and are available at www.addgene.org. **d** Timeline for production of recombinant baculovirus using the FlexiBAC system. On day 1, the user transfects Sf9 insect cells with pOCC shuttle vector (with target gene of interest) and linearized DefBac DNA. Within some insect cells, pOCC and DefBac will recombine and this event generates an infectious baculovirus that propagates throughout the culture. On day 5, the user collects the conditioned media (which contains released baculovirus) and uses it to infect fresh Sf9 insect cells. On day 10, the user uses the conditioned media again to infect fresh Sf9 cells. On day 13, the user harvests the infected Sf9 cells containing the target protein of interest (represented by green, GFP+ cells). For secreted protein targets the conditioned medium is collected after this phase

To streamline the production of viral stocks, we engineered a bacmid encoding a replication-defective baculovirus, which we term “DefBac” (Fig. 1). This bacmid is designed to recombine with one of 143 compatible shuttle vectors, called pOCC vectors, where the target gene of interest (GOI) is inserted (see Additional files 1 and 2). Transfection of *Spodoptera frugiperda* (Sf9) insect cells with linearized DefBac and a pOCC vector permits their recombination and produces infectious baculovirus. The GOI is then expressed late in the lytic cycle using the polydedrin promoter. Viruses are produced and escape the Sf9 cells only if recombination between pOCC and DefBac was successful. One round of viral amplification is typically required before the virus is used to express the target protein. The general workflow is shown in Fig. 1d; a detailed, step-by-step protocol is included in the supplement (see Additional file 3). In the following sections, we describe construction of DefBac and the pOCC shuttle vectors.

### A doubly defective viral backbone ensures production of complete recombinant viruses containing the gene of interest

One of the earliest, and still very popular, systems used to generate recombinant baculovirus for protein expression (Bac-to-Bac®, Thermo Fisher Scientific) employs site-specific transposition of a baculovirus genome, propagated as a bacmid, with a cloning plasmid that contains the gene of interest. Transposition occurs in bacteria, which can take ~ 1 week to select and isolate positive clones. To streamline recombinant virus preparation, we modified the *Autographa californica* baculovirus bacmid (bMON14272) by deleting the 3′-ends of two essential genes: *lef-2* and *AcOrf-1629*. These genes flank the polyhedrin locus and are required for baculovirus production in cultured insect cells [8–11]. Cells transfected with this defective bacmid never appeared infected with baculovirus, and the cells continued to grow and divide at a rate similar to non-transfected controls (data not shown). In contrast, insect cells transfected with the unmodified parental bacmid produced infected cells 72 h post transfection, as expected. We refer to this doubly defective bacmid as H092. The design ensures that the parental bacmid lacking the gene of interest will not produce functional baculovirus.

**Fig. 2:**
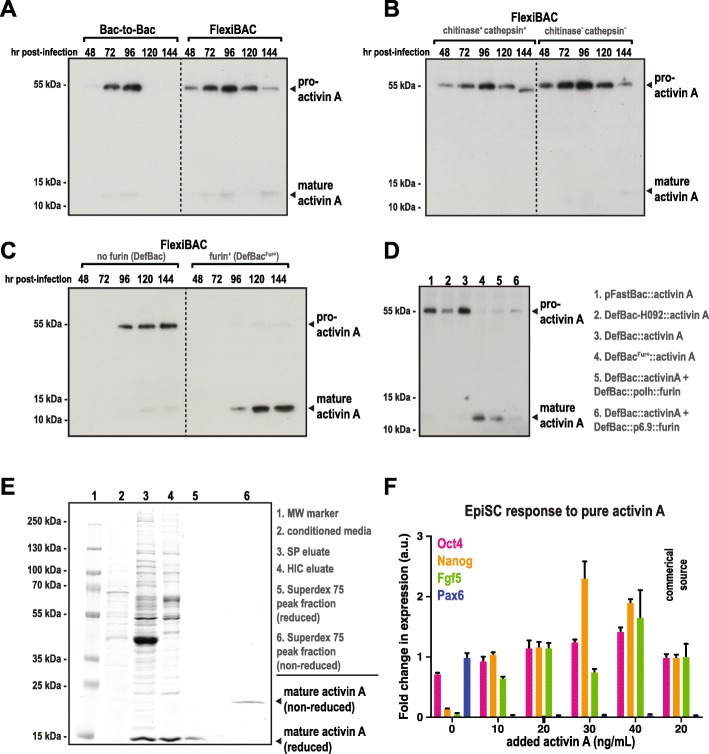
Efficient production of mature TGF-β member Activin A using FlexiBAC. **a** Sf9 insect cells were infected with recombinant baculovirus generated using the commercial Bac-to-Bac system (ThermoFisher Scientific) or the FlexiBAC system. Cell supernatants were collected at the indicated times after infection, resolved on 4–20% gradient SDS-PAGE gels under reducing conditions, then analyzed by western blot with anti-Activin A IgG. Bands corresponding to pro-Activin A (uncleaved) and mature Activin A (cleaved) are indicated. **b** Activin A secretion from insect cells infected with baculovirus generated using the precursor backbone for the FlexiBAC system (called “DefBac-H092”, which expresses viral chitinase and cathepsin) or the current backbone (called “DefBac”, which does not express viral chitinase and cathepsin). **c** Activin A maturation and secretion from insect cells infected with DefBac-derived baculovirus vs. DefBac^Fur+^-derived baculovirus. In addition to expressing the target protein of interest, DefBac^Fur+^ expresses furin convertase, which converts pro-Activin A to its mature form. **d** Insect cells were either singly infected or co-infected with the following virus: pFastBac::Activin A (lane 1), DefBac-H092::Activin A (lane 2), DefBac::Activin A (lane 3), DefBac^Fur+^::Activin A (lane 4), DefBac::Activin A and DefBac::polh::Furin (lane 5), DefBac::Activin A and DefBac::p6.9::Furin (lane6). Each virus was added to insect cells at MOI = 0.2 except for DefBac::p6.9::Furin (MOI = 2). The conditioned media was analyzed by western blot. Samples taken from peak Activin A expression times are shown (96 hpi for conditions 1–5, 72 hpi for condition 6). **e** Multi-step purification of mature Activin A from conditioned media from insect cells infected with DefBac^Fur+^::Activin A. Samples were resolved on an SDS-PAGE gel and stained with coomassie blue. Samples 1–5 were reduced with DTT prior to loading. **f** To assess activity, purified mature Activin A was added to epiblast derived stem cells (EpiSCs) cultured on fibronectin. Fold changes in gene expression, normalized to beta-actin, were determined over 11 days by quantitative polymerase chain reaction (qPCR) for the pluripotency makers Oct4, Nanog, Fgf5, and for the lineage marker, Pax6 (mean +/− S.D.; *n* = 3 separate experiments). Activin A from a commercial source was used as a control (grey box)

We designed H092 so that infectivity is restored by site-specific recombination with a pOCC shuttle vector, which contains the gene of interest flanked by 3′-segments of *lef-2* and *AcOrf-1629* that complement the truncations in H092 (Fig. 1a). Recombination at the *lef-2* and *AcOrf-1629* loci restores functionality of these genes, thus producing an infective, recombinant baculovirus genome that expresses the gene of interest (Fig. 1a). Sf9 insect cells efficiently promote homologous recombination, suggesting that co-transfection of H092 and pOCC in Sf9 cells could reliably produce recombinant bacmid and functional virus. Indeed, co-transfection of H092 and a shuttle vector expressing GFP, under the control of the polyhedrin promoter, produced infected, GFP-positive Sf9 cells four days post-transfection (see Fig. 2 for more examples).

To demonstrate that GFP expression was associated with the production of an infectious virus, we collected the conditioned media from transfected cells and diluted it into fresh cultures. After five days, these cultures were incubated with fluorescently-tagged antibody that recognizes the baculovirus GP64 coat protein on the plasma membrane of infected insect cells and then single cell FACS sorted. These singly sorted cells were then added to fresh cultures in a 96-well plate. Out of 96 wells, 34 wells contained clearly infected cells that also strongly expressed GFP. The remaining 62 wells showed neither signs of infection nor GFP expression. These results show that rescue was both precise and efficient and that only recombinant virus expressing the transgene supplied by the shuttle vector are replication-competent. This design ensures that the parental bacmid lacking the gene of interest will not produce functional baculovirus.

### Deletion of two non-essential baculovirus genes improves protein expression

Proteins required for baculoviral host liquefaction are not necessary for infection in cell culture and can often interfere with target protein expression. Two examples include cathepsin (*v-cath*) and chitinase (*chiA*), which are abundantly secreted late in the lytic cycle. Kaba et al. (2004) demonstrated that deletion of these genes improved the integrity and yield of a secreted target protein, p67. To improve our system, we eliminated these genes in H092. The resulting bacmid, called DefBac, produced infective baculovirus when co-tranfected with a pOCC shuttle vector and enhanced protein expression of the TGF-β member Activin A (Fig. 2a; see below).

### A versatile shuttle vector set enables combinatorial appending of tags to the target protein

It is often desirable to append different tags to the target protein to improve expression levels, solubility, purity and visualization. To facilitate this optimization process, we developed a compatible set of 143 shuttle plasmids, called Optimized Classic Cloning (pOCC) vectors, designed to recombine with DefBac and express the user’s GOI (Fig. 1b). Using this system, one GOI can be easily inserted into numerous vectors to produce target proteins with various combinations of cleavable N- and C-terminal tags (Fig. 1c). The Defbac viral genome and the most popular 52 pOCC shuttle vectors are available at www.addgene.org (see Additional file 2). The remaining pOCC shuttle vectors are available upon request (see Additional file 1 for the complete collection).

Each pOCC vector contains the same cloning cassette demarcated by NotI and AscI restriction enzyme sites. This cassette encodes a constitutive promoter (gb3) driving the toxic *ccdB* gene (Fig. 1b). A pOCC vector with no insert will kill ccdB-sensitive bacteria (e.g. DH5α) and therefore must be maintained in a resistant strain such as DB3.1. When the target GOI is successfully inserted between the NotI and AscI sites, the *ccdB* gene is lost and the plasmid can be maintained in ccdB-sensitive cells. This feature dramatically reduces isolation of vectors lacking insert (data not shown). If target GOIs contain internal sites for NotI and AscI enzymes, their removal by silent mutation is required. However, recognition sites for both enzymes are 8 bp in length; thus, in practice, this additional mutagenesis step is rarely needed.

On the 5′ and 3′ ends of the cloning cassette are modules encoding purification tags, fluorescent probes, conjugating tags, trafficking peptides, and cleavable linkers (Fig. 1c; see Additional file 1 for a full list and description of tags)*.* The translational reading frame is adjusted so that the NotI and AscI cloning sites encode amino acids with small side chains often found in natural peptide linkers. Upstream of these sequences lie a polyhedrin promoter and a translational start site, while stop codons and transcriptional terminator elements lie downstream (Fig. 1b). Once a GOI is synthesized with compatible NotI and AscI ends, it can be inserted or subcloned into pOCC vectors to produce in-frame fusion proteins.

### An enhanced version of DefBac that highly expresses a pro-protein convertase

Many secreted proteins require pro-protein convertases to generate the functional, mature form. We hypothesized that high expression of such target proteins might overwhelm the endogenous proteolytic processing enzymes in the Golgi apparatus. To elevate furin protein levels, we replaced the conotoxin gene in DefBac with full-length cDNA encoding murine furin driven by the p6.9 promoter. This promoter directs transcription earlier during the lytic cycle compared to the strong polyhedrin (polh) promoter driving target gene expression [12]. Thus, increased levels of furin are present before the target is translated. We call this parental bacmid “DefBac^Fur+^”.

### Expression and secretion of mature TGF-β family proteins

Transforming growth factor Beta (TGF-β) family proteins are secreted proteins involved in inter-cellular communication during development and tissue homeostasis. After translation, these proteins are imported into the ER in a pro-form, then trafficked to the Golgi apparatus, where they undergo maturation through proteolytic cleavage by furin convertases. The mature form is then secreted to the extracellular space by exocytosis. Recombinant TGF-β proteins are difficult to express at high levels in baculovirus-infected insect cells due to limited capacity of the secretory pathway. To overcome this limitation, we employed our enhanced version of DefBac, called DefBac^Fur+^, which expresses furin.

We focused on production of recombinant Activin A, a secreted TGF-β protein involved in inflammation, neural development, and hematopoiesis [13, 14]. We co-transfected insect cells with a pOCC plasmid (pOCC-RL) containing Activin A and parental bacmid, then collected conditioned media containing secreted protein for 6 days post infection (dpi). We achieved higher Activin A yields using the DefBac parental bacmid compared to a conventional bacmid used in commercially-available systems (bMONH092, the bacmid encoding the baculvirus genome in the Bac-to-Bac system) (Fig. 2a). This improvement is due to the elimination of genes encoding cathepsin and chitinase, two secreted proteins that are only required for host liquefaction (Fig. 2b). However, the majority of Activin A was in the inactive pro-form (55 kDa); only trace amounts of the active, mature Activin A form (13 kDa) appeared (Fig. 2a, b; reducing conditions).

We next sought to improve production yields of mature Activin A. To become fully active, TGF-β -family proteins require C-terminal cleavage by furin convertases that reside in the early Golgi apparatus and recognize the multi-basic motif RX(K/R)R [15]. Western blot analysis revealed that DefBac^Fur+^ greatly improved the production of mature Activin A and its secretion into the extracellular medium, with levels peaking 120 hpi (Fig. 2c). We detected only trace amounts of the pro-form of Activin A in the DefBac^Fur+^ sample (Fig. 2c). DefBac^Fur+^ also produced more mature Activin A compared to cultures co-infected with virus expressing Activin A and virus expressing furin (Fig. 2d).

Next, we purified this mature Activin A and determined its activity. We isolated Activin A from the conditioned medium by cation exchange chromatography (SP), followed by hydrophobic interaction chromatography (HIC) and size exclusion chromatography (Superdex 75). From 1 L of conditioned medium, 0.5 mg of pure Activin A was obtained (Fig. 2e). Addition of purified Activin A (from 10 to 40 ng/mL) to epiblast-derived stem cells (epiSC) suppressed expression of the differentiation marker Pax6 and either maintained or potently promoted the expression of pluripotency markers such FGF5, Nanog, and Oct4, indicating that Activin A was active (Fig. 2f).

**Fig. 3:**
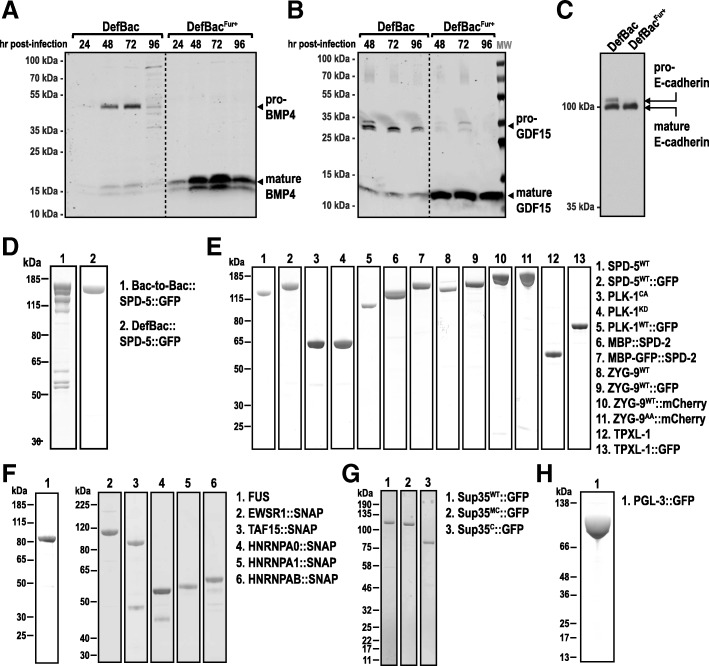
Production of additional recombinant proteins using FlexiBAC. **a** Human Bone morphogenetic protein 4 (BMP4) was expressed in insect cells using the two different FlexiBAC viral backbones that differ in their expression of recombinant furin: DefBac and DefBac^Fur+^. BMP4 levels in the conditioned media were analyzed by western blot using an anti-BMP4 antibody. **b** Human Growth/differentiation factor 15 (GDF15) was expressed in insect cells using DefBac and DefBac^Fur+^. GDF15 levels in the conditioned media were analyzed by western blot using an anti-GDF-15 antibody. **c** The ecto-domain of canine E-cadherin was expressed in insect cells using DefBac and DefBac^Fur+^. E-cadherin levels in the conditioned media were analyzed by western blot using an anti-E-cadherin antibody. **d** Coomassie-stained gels depicting 6xHis-tagged SPD-5::GFP expressed with the Bac-to-Bac system (ThermoFisher Scientific) or with DefBac. In both lanes, SPD-5::GFP was purified from insect cell lysate using Ni-NTA affinity chromatography, then eluted with 250 mM imidazole. **e** All proteins in (B-E) were expressed in SF9 insect cells using the FlexiBAC system. Coomassie-stained gels depicting purified *C. elegans* centrosome proteins. For more information, see [17, 27]. **f** Coomassie-stained gels of purified human proteins that contain long stretches of intrinsically-disordered regions. For more information, see [22, 25]. **g** Coomassie-stained gels depicting purified yeast prion-like proteins. MC and C represent middle and C-terminal domains of Sup35. For more information, see [24]. **h** Coomassie-stained gel of purified PGL-3, a protein that constitutes P granules in *C. elegans* embryos. For more information, see [23]

The TGF-β family represents a broad class of proteins that include growth, differentiation, and morphogenetic factors. In addition to Activin A, two prominent examples are human growth/differentiation factor 15 (GDF15) and human bone morphogenetic protein 4 (BMP4) [16]. Expression of these targets using DefBac^Fur+^ greatly improved their processing and secretion levels (Fig. 3a and b). Thus, DefBac^Fur+^ is generally suited for recombinant production of active forms of secreted TGF-β proteins.

### Expression and secretion of processed E-cadherin

To test whether other secretory protein families similarly benefit from co-expression with furin, we generated recombinant baculovirus to express the ectodomain of canine E-cadherin, which requires maturation of the pro-form by a furin-like convertase. When the E-cadherin ectodomain is produced from DefBac virus, some unprocessed, pro-form is secreted into the conditioned medium (Fig. 3c, lane 1) However, when we used DefBac^Fur+^, only the mature, fully-processed ectodomain was secreted (Fig. 3c, lane 2). We conclude that DefBac^Fur+^ is suitable for production and secretion of a range of secreted proteins that require furin-dependent processing.

### Expression of elongated, coiled-coil centrosome proteins

Centrosomes are micron-scale organelles that nucleate and organize microtubules needed for mitotic spindle assembly. Centrosomes comprise barrel-shaped centrioles surrounded by a less structured mass of protein called pericentriolar material (PCM). Mechanistic understanding of how PCM assembles and nucleates microtubules has long been hampered by lack of in vitro reconstitution approaches. This is attributed to the PCM scaffold being composed of elongated, high-molecular weight proteins that contain coiled-coil domains, which have been notoriously difficult to reconstitute in their full length.

The main PCM scaffold protein in the nematode *C. elegans*, SPD-5, contains 9 predicted coiled-coil domains, has a molecular weight of 135 kDa, and a hydrodynamic radius of ~ 8 nm [17, 18]. Compared to the Bac-to-Bac system (ThermoFisher Scientific), use of the FlexiBAC system improved the consistency and yield of full-length SPD-5 (Fig. 3d). We then used FlexiBAC to express and purify 12 additional full-length PCM proteins (Fig. 3e), which were sufficient to assemble minimal PCM and nucleate microtubule asters in vitro [17]. Thus, the FlexiBAC system is suited for expressing elongated coiled-coil proteins, as well as globular kinases and microtubule-stabilizing enzymes.

### Expression of intrinsically-disordered proteins that self-assemble

Approximately 20% of the proteome in eukaryotes comprises protein sequences that lack globular structure, termed “intrinsically-disordered” proteins (IDPs) [19]. Numerous non-membrane-bound organelles—such as nucleoli, stress granules, and germ granules—assemble through the coalescence of IDPs through multivalent interactions with themselves, other IDPs, or RNAs [20, 21]. Historically, IDPs have been difficult to reconstitute, as they are prone to aggregation and end up in inclusion bodies.

The FlexiBAC system was successful in expressing full-length IDPs that form the basis of stress granules (in human cells and yeast) and germ granules (in *C. elegans*) (Fig. 3f-h). Target proteins were isolated from infected cells after multi-step purifications in sufficient yields (1–100 mg/L culture) to carry out extensive biochemical reconstitution assays. Under specific buffer conditions, each of these proteins self-assembles into micron-scale condensates [22–25]. We conclude that FlexiBAC is suitable for expression of disordered proteins that exhibit a strong tendency to self-assemble.

## Discussion

Baculovirus expression has long been used for generating high yields of eukaryotic proteins that are properly folded and have native post-translational modifications. To make baculovirus expression simpler, more reliable, and more flexible, we generated a new system called FlexiBAC. We have demonstrated that FlexiBAC can be used to express diverse targets, including secreted proteins that require proteolytic processing in the Golgi apparatus, as well as intrinsically disordered and coiled-coil proteins.

FlexiBAC exhibits a number of benefits. First, the time from initial cloning to protein production can be as little as 13 days. Second, FlexiBAC is versatile and amenable for screening, as we designed a compatible shuttle vector set (143 vectors) harboring numerous tags used for purification, visualization, trafficking, and labeling with chemical probes. The target gene of interest is easily swapped between the plasmids using classic restriction enzyme cloning techniques. Third, FlexiBAC is suitable for expression of secreted proteins and is ideal for proteins requiring processing by the Golgi-resident furin convertase. Using a viral backbone that overexpresses furin, called DefBac^Fur+^, we produced high yields of Activin A, a TGF-β family protein. We expect our system to be useful for expressing mature versions of other targets requiring processing by a pro-protein convertase. Finally, the FlexiBAC system is open-source and freely available through www.addgene.org.

FlexiBAC has been useful for expressing difficult proteins in numerous studies [17, 22–30]. We envision additional modifications to specifically address other classes of protein targets. For example, with proteins found in heteromeric complexes, expression of individual subunits often is unsuccessful but is greatly improved by stoichiometric co-expression of all the subunits. We are currently adapting the FlexiBAC system to co-express multiple genes for stoichiometric expression of all subunits [31]. In addition, FlexiBAC could also be expanded to express additional factors necessary for native post-translational modifications such as other pro-protein convertases, kinases, phosphatases, and glycosylation enzymes. Use of the DefBac^Fur+^ bacmid might also improve maturation of recombinant proteins secreted by baculovirus-infected insect larvae. Larval expression produces very high levels of secreted recombinant proteins and is more economical compared to expression in cultured insect cells [32, 33]. Thus, use of FlexiBAC might be applied for large-scale production of growth factors or nanobodies.

The speed, simplicity, and flexibility of the FlexiBAC system make it an ideal starting platform for expression and screening of diverse types of recombinant proteins. Given that FlexiBAC is open-source, we invite improvements that will expand its applicability and facilitate production of a broader range of proteins.

## Conclusions

FlexiBAC is an open-source baculovirus expression system that is freely available at www.addgene.org. The FlexiBAC system includes a shuttle vector set that can append 143 combinations of tags to the user’s protein of interest. Finally, FlexiBAC is engineered to permit high level expression of secreted proteins that require furin-dependent proteolytic maturation. By increasing protein tagging possibilities, allowing bacmid formation within insect cells, and providing selection against improper baculoviruses, FlexiBAC simplifies baculovirus-mediated expression of cytosolic proteins and secreted proteins that require proteolytic processing.

## Methods

### Reagents

Restriction enzymes were purchased from New England Biolabs (Ipswich, MA, USA). *E. coli* DH10Bac containing bMON14272 [34] was purchased from Thermo Fisher Scientific (Waltham, MA, USA). *E. coli* GB2005 and pSC101-BAD-gba-Cm [35] were kindly provided by Francis Stewart (Tech. Univ. Dresden). Colicin E1 was expressed and purified as a His6-tagged fusion from pETMM11_colE1_imm, an expression construct derived from pT7-7_ColE1/Imm that was kindly provided by William A. Cramer (Purdue Univ.). pOET1, pBAC4X-1, the human activin A gene in pCMV6-AC (SC319183) were purchased from Oxford Expression Technologies (Oxford, UK), EMD Millipore (Billerica, MA, USA), and OriGene (Rockville, MD, USA) respectively. DNA constructs and maps were designed using Gene Construction Kit 3.5 from Textco Biosoftware, Inc. (West Lebanon, NH, USA). pSVL fur-mur (D1352) containing murine furin was a kind gift from L. Robertson (Univ. of Oxford) [36]. Sf-9ET cells developed by Hopkins and Esposito (NCI, Bethesda, MD) [37] for baculovirus titering were kindly provided by Sabine Suppmann (MPI-Biochemie, München). Sf9 ESF insect cells and ESF 921 Insect Cell Culture Medium, Protein-Free, were purchased from Expression Systems (Davis, CA, USA). Escort™ IV transfection reagent was purchased from Sigma-Aldrich (St. Louis, MO, USA). Oligonucleotides were purchased from Metabion (Planegg, Oberbayern, Germany). Infected insect cells were sorted using the BD FACS Aria cell sorter from BD Biosciences (San Jose, CA, USA).

### Generation of the double defective baculovirus genome (DefBac) using recombineering

We used the selection/counterselection system employing TolC, an *E. coli* outer membrane protein, in combination with the λ redgam recombination system to generate seamless insertions or deletions in both a bacmid encoding the baculovirus genome as well as the *E. coli* genome [38]. This method generates rapid and “scar free” modifications and avoids the use of classic antibiotic drug selections

Prior to carrying out recombineering on the baculovirus bacmid, the genomic copy of TolC was deleted from the host *E. coli* strain, GB2005. This deletion was made by inducing Lambda Red recombination enzymes using pSC101-BAD-gba-Cm followed by transformation with an oligo (B22I3; all oligonucleotides sequences are listed in Table S1 in Additional file 1), homologous to genomic sequences flanking the TolC gene. Correct clones were identified as having resistance to the toxin Colicin E1. This deletion strain, named RL001, was sensitive to sodium dodecyl sulfate (SDS) and could host seamless TolC selection/counterselection recombineering of the bacmid encoding the baculovirus genome (bMON14272).

To generate baculovirus bacmid that could not self-replicate, we truncated two genes, *lef2* and Ac-*Orf1629*, which flank the *polyhedrin* locus using Lambda Red recombineering in RL001. For each deletion, the C-terminus of the encoded protein was replaced with a cassette composed of the TolC gene flanked with 50 base pair homology arms for precise integration into the target DNA. Subsequently, the TolC gene was removed by counterselection after recombination of the bacmid with an oligonucleotide composed of a direct fusion of those same homology arms. The strain harboring the bacmid with these two disabled baculovirus genes was named H092.

TolC recombineering was then used to delete *v-cathepsin* and *v-chitinase* genes from the H092 bacmid to generate H099, which is the final baculoviral genome we termed “DefBac”. More detailed information about intermediate steps of bacmid production can be found in the supplement.

### Generation of the pOCC shuttle vectors

We modified the vector pOET1 (Oxford Expression Technologies) following the Uracil-Specific Excision Reagent (USER) cloning technique (New England Biolabs). Briefly, the vector backbone of EcoRI linearized pOET1 was amplified by PCR using primers B28B9 and B28C1. Primers B22F6 and B22F7 were then used to amplify the polyhedrin promoter/MCS/polyA terminator cassette of EcoRV linearized pFastBacM11. The two PCR products were then treated with the USER enzyme and transformed into DH5α cells to generate the desired construct, pOEM1.

To make the first pOCC baculovirus shuttle vector, pOCC5, a PCR fragment containing the *ccdB* gene under the constitutive *gb3* promoter was amplified from the vector pNCP2 [39] with primers to generate NcoI and HindIII ends. This fragment was cut with NcoI and HindIII and then sub-cloned into pOEM1. We then inserted sequences for both N- or C-terminal tagging (see Fig. 1). In the course of this a work a diverse collection of baculovirus shuttle vectors was generated in this manner (see Additional file 1). All the pOCC shuttle vectors and defective bacmids described herein are available either at www.addgene.org or upon request. Detailed information about construction of intermediate plasmids and viral backbones can be found in the supplement.

### Antibodies

Goat anti-Activin (#AF338) and Goat anti-BMP4 (#AF757) antibodies were purchased from R&D Systems (Minneapolis, MN, USA). Rabbit anti-Furin (#PA1–062) antibody was purchased from Thermo Fisher Scientific (Waltham, MA, USA). PE labeled Mouse anti-GP64 antibody (#sc-65,498 PE) was purchased from Santa Cruz Biotechnology (Dallas, TX, USA). Mouse Penta-His antibody (#34660) was purchased from Qiagen (Hilden, Germany). Rabbit anti-GDF15 (Mic-1/D2A, #8479; Rabbit mAb) was purchased from Cell Signaling.

### Cell culture

Sf9-ESF insect cells (Expression systems, Davis, CA, USA) were cultured at 27 °C in suspension in glass culture flasks in ESF 921 serum-free medium (Expression Systems, Davis, CA, USA).

### Western blot analysis

After infections, the supernatant samples are resolved into reducing 4–20% gradient SDS–PAGE gels. Cell lysates are prepared with PBS buffer supplemented with a cocktail of protease inhibitors. Separated proteins are then transferred onto nitrocellulose membranes, blocked for 2 h, and probed overnight with Goat anti-Activin IgG (1:5000) (R&D systems, Minneapolis, MN), Rabbit anti-Furin IgG (1:2000) (Thermo Scientific) or Goat anti-BMP4 (1:5000) (R&D systems). Immunoreactive bands are revealed using ECL detection.

### Activin a activity assay

Epiblast derived stem cells (EpiSCs) were cultured on fibronectin in N2B27 medium with 12 ng/ml FGF2 and the respective amount of ActA for 11 days over 4 passages. Fold changes in gene expression, by quantitative polymerase chain reaction (qPCR), were determined for cells for the pluripotency makers Oct4, Nanog, Fgf5, and for the lineage marker, Pax6 and normalized to levels of beta-actin. Results are presented as the mean of triplicate determinations with error bars showing the standard deviation.

## Additional files

Additional file 1: **Table S1.** Inventory of available pOCC shuttle vectors. The user’s gene of interest can be inserted into 143 available vectors using NotI and AscI restriction cloning (XLSX 14 kb)

Additional file 2: **Table S2.** Inventory of pOCC shuttle vectors available at www.addgene.org. The 52 most popular shuttle vectors were deposited to Addgene (XLSX 11 kb)

Additional file 3: Supplementary Materials and Methods. A detailed protocol for using FlexiBAC and more information on plasmid and bacmid construction. (DOCX 39 kb)

## Abbreviations

dpi: Days post infection
EpiSCs: Epiblast derived stem cells
FACS: Fluorescence activated cell sorting
GFP: Green fluorescent protein
GOI: Gene of interest
IDP: Intrinsically disordered protein
MOI: Multiplicity of infection
Sf9: *Spodoptera frugiperda* cell line
TGF-β: Transforming growth factor Beta
