# Supplementary material for: FlexiBAC: a versatile, open-source baculovirus vector system for protein expression, secretion, and proteolytic processing
Source: BMC Biotechnol. 2019 Mar 29;19:20. doi: 10.1186/s12896-019-0512-z (PMC6441187; doi:10.1186/s12896-019-0512-z)
Supplement: Supplementary file 3 — Supplementary Materials and Methods. A detailed protocol for using FlexiBAC and more information on plasmid and bacmid construction. (DOCX 39 kb) [file 12896_2019_512_MOESM3_ESM.docx]

**SUPPLEMENTAL MATERIALS AND METHODS**

**FlexiBAC Protocol**

**Reagents**

- 24-well round-bottom plates (Qiagen #19583)
- SF9-ESF insect cells (Expression Systems # 94-001F)
- ESF921 Insect cell culture medium (Expression Systems #96-001-01)
- Autoclaved 2mL tubes
- Breathe-Easy sealing membranes (Sigma #Z380059-1PAK)
- 0.45 μm PVDF syringe filters
- 10 ml and 60 ml syringes
- Pen-Strep (Gibco)
- Fetal Bovine Serum (Gibco)
- Escort IV transfection reagent (Sigma #L-3287)
- DNA isolation kit (Macherey-Nagel; Nucleobond Xtra Maxi 740414.50)
- SbfI-HF (NEB R3642-S)

**Prep Phase 1. Make a stock of the DefBac viral backbone.**

1. Inoculate 1 L LB media with DefBac transformants (in DH10B cells). Add 30 mg Kanamycin (30 μg/ml final concentration). Incubate overnight until stationary phase.
2. Harvest bacteria, split into two tubes. Isolate DefBac using a maxi prep DNA isolation kit. Stop at the 70% ethanol wash step.
3. IMPORTANT: from here on, work in a sterile hood. Decant the final 70% ethanol wash. Do not dislodge the DNA pellet.
4. Air dry DNA (5-10 min). Do not over-dry the pellet.
5. Reconstitute DNA with 500 µl TE (sterile). Just add the buffer and let the DNA dissolve overnight at 4°C. Then mix by gentle pipetting (you want to avoid shearing).
6. Measure concentration of DNA by determining the absorbance at 260 nm (e.g. using a NanoDrop).
7. Digest with SbfI-HF enzyme to linearize DefBac. Incubate 3 hr at 37°C, then 20 min at 65°C to inactivate the SbfI.

32 µl dH20

30 µl DefBac DNA (15-30 µg)

1 µl SbfI-HF

7 µl CutSmart Buffer

___

70 µl

1. Determine the proper amount of the linearized DefBac DNA using transfection with a control plasmid (pOCC5; this expresses eGFP). For example, test 4, 2,1, 0.5, and 0.25 µL of SbfI-digested DefBac DNA, following the steps described in Production Phase 1, Day 1.

**Prep Phase 2. Subclone target gene of interest into pOCC vector and miniprep**

1. Use NotI and AscI restriction enzymes to digest pOCC vector and DNA encoding the target gene of interest.
2. Ligate plasmid and inserts, transform into DH5alpha cells, and grow cells overnight on LB + ampicillin plates.
3. Pick positive transformants and grow overnight cultures. Isolate DNA using a mini prep kit.

**Production Phase 1. Transfect SF9 cells with pOCC plasmid and DefBac viral backbone to make the P1 virus stock.**

*A few days before transfection:*

Grow up an appropriate amount of SF9 insect cells. We typically passage cells on Friday morning to 0.5 x 10^6^ cells/ml and let them grow to 4-5 x 10^6^ cells/ml, which is usually by Monday morning. This culture is directly used for transfection.

*Day 1:*

1. Spray the 24-well plates with 70% ethanol and air dry in the hood.
2. Prepare transfection mix. In each well add reagents in the following order:

800 μl Insect cell medium

12 μl Escort IV transfection reagent

1 μl SbfI-digested DefBac DNA**

1 μl pOCC plasmid (0.1 µg/µL)

__

814 μl

**This amount should be optimized for each new batch of DefBAC DNA.

1. Gently mix the plate by shaking and incubate under the hood for 15 min. Add 200 μl cells per well (5 x 10^6^ cells/ml stock; this will result in 1 x 10^6^ cells/ml cells in the final mix).
2. Cover plate with a Breathe-Easy membrane.
3. Seal membrane/plate boundary using parafilm.
4. Place the sealed plate in an incubated shaker (200 rpm, 27°C).

*Day 2:*

1. Make master mix of insect cell medium + 1:50 Pen-Strep + 4% Fetal Bovine Serum (FBS). Note: addition of serum is optional but is advised for long term storage

18.8 ml insect cell medium

800 µl FBS (100% stock)

400 µl Pen-Strep

1. ml
2. Take out the sealed plate from the incubated shaker. In a sterile hood, add 1 ml of master mix to each well that contains insect cells. This will result in final concentrations of 1% Pen-Strep and 2% FBS.
3. Cover plate with Breathe-Easy membrane and seal the edges of the membrane with parafilm. Place the sealed plate in a shaking incubator (200 rpm, 27°C).

*Day 5:*

1. Gently spin the plates at 500xg for 5 min at room temperature.
2. Recover supernatant (1.5 ml). This contains released virus, called P1 virus.
3. Store P1 virus at 4°C protected from light.  The P1 virus can also be frozen using a cryoprotectant in a slow cooling device as per freezing cultured cells.

**Production Phase 2. Amplify virus to get P2 stock.**

1. A few days prior, prepare an appropriate amount of insect cells so that they will be in log phase and at a density of 0.5 X 10^6^ cells/ml on the day of infection.
2. Add 50 µl P1 virus to 50 ml cells in a 250 ml flask. Add FBS (2% final concentration) and Pen-Strep (1% final concentration). (Note: addition of serum is optional but is advised for long term storage)
3. Shake for 5 days in an incubated shaker (100 rpm, 27°C).
4. Spin down cells at 2000xg for 5 min at room temperature. Collect supernatant and pass through a syringe filter. This is the P2 virus stock.
5. Store P2 virus at 4°C protected from light.  The P2 should be good for ~1 month.

**Production Phase 3. Express target protein and harvest cells.**

1. Infect log phase insect cells (1 X 10^6^  cells/ml) with virus. In the beginning, try a 1:100 dilution of P2 virus; e.g., add 5 ml virus for 500 ml insect cells. If optimization is necessary, try a dilution series.
2. Shake infected cells for 72 hr (100 rpm, 27°C).  Note: to determine the optimal time of harvesting, we advise performing a time course of expression. Collect samples at 48, 72, 96 hours post infection and determine the level of expression of the target protein by SDS-PAGE and western blot.
3. Harvest cells using a gentle spin and a slow deceleration (300xg for 10 min).
4. Resuspend cell pellet with an appropriate buffer (no detergent!) and transfer to several 14 ml Falcon tubes.
5. Flash freeze Falcon tubes in liquid nitrogen. Store at -80°C. Note: some proteins are prone to degradation after freeze/thaw. In this case, it is advisable to proceed with extract preparation and purification using the fresh cell pellet.

**Detailed information about bacmid and plasmid construction**

pBSKS-TolC (D1495)

A cassette containing the tolC gene with promoter, to serve as a selection/counter-selection marker for recombineering, was ampliﬁed by PCR from strain GB2005 with primers B22A2 and B22A3 (tolC_cassette0, Table S1). The PCR product was inserted into pBlueScriptKS- between the BamHI and NotI to generate D1495.

pFastBacM11Activin A

The hActivin A (Origene, D1661) was sub-cloned between the NcoI and XhoI sites in pFastBacM11.

pOEM1

We modified the vector pOET1 (Oxford Expression Technologies) following the Uracil-Specific Excision Reagent (USER) cloning technique (New England Biolabs). The vector backbone of EcoRI linearized pOET1 was amplified by PCR using primers B28B9 and B28C1. Primers B22F6 and B22F7 were then used to amplify the polyhedrin promoter/MCS/polyA terminator cassette of EcoRV linearized pFastBacM11. The two PCR products were then treated with the USER enzyme and transformed into DH5α cells to generate the desired construct, pOEM1.

pOEM1ActivinA construct

The human Activin A gene (OriGene) was sub-cloned between the NcoI and XhoI sites in pOEM1.

pOEM1Furin

Furin was amplified from pSVL fur-mur (Constam and Robertson, 1999) using primers B30D9 and B30E1 and then joined by USER sub-cloning to the vector backbone of EcoRI linearized pOEM1 PCR amplified using B28H9 and B28I3.

pOEM5

The polyhedron promoter in pOEM1 was replaced with the p6.9 promoter to generate pOEM5. This was carried out by PCR amplification of EcoRI linearized pOEM1 with primers B28F8 and B28B9. This PCR product was then joined by USER cloning to that generated from the pOEM1 plasmid amplified with primers B28F7 and B22F7.

pOEM5Furin

Furin was amplified from pSVL fur-mur (D1352) with primers B30D9 and B30E1 and then fused via USER cloning to the PCR product generated with primers B28F8 and B28H9 using NcoI/XhoI linearized pOEM1 as template.

pCR2.1p6.9Furin (D1670)

This construct, placing the p6.9 promoter upstream of mouse Furin, was amplified from pSVL fur-mur (D1352) with the primers B29D3, which contains the p6.9 promoter sequence, and B29I7 and then TOPO cloned into pCR2.1 to obtain pCR2.1p6.9Furin (D1670).

pBSKS tolC_rpsL (D1668)

The *tolC* gene was amplified from D1495 with primers B30A9 and B30B1. The rpsL gene was amplified from pBeloBAC11-epo-oriT-trpE-ta-pm-no-epoK-apra-Rpsl-amp, a kind gift from Mihail Sarov (MPI-CBG, Dresden) with primers B30B2 and B30B3. These products were mixed, fused by five cycles of overlap PCR then amplified after addition of primers B30A9 and B30B3. After digestion with NotI and BamHI, this PCR product was ligated into pBlueScript KS.

pCR2.1p6.9furin_tolC_rpsl (D1678)

This construct served as the DNA template to obtain the tolC-furin_cassette for insertion into the H131 bacmid. The rpsL gene was included in this construct but was not, in the end needed for selection with streptomycin. The tolC-rpsL cassette was amplified from D1668 with the primers B30D6 and B30D7. Primers B30C7 and B30C8 were used to PCR amplify the backbone of pCR21p6.9Furin (D1670) and this was joined to the tolC-rpsL cassette by USER cloning.

pBAC4x-1BMP4

Human BMP4 was cloned into pBAC4x-1, under the polyhedrin promoter.

pOEM1_GDF15

H. sapiens GDF15 was amplified by PCR from pENTR223 GDF15 (a kind gift from Jui-Hsia Weng) and cloned into pOEM1 using BspHI and HindIII.

pOCC_E-cadherin_ecto

A gene encoding the ecto domain of human E-cadherin fused to N-terminal GFP and SNAP tags was synthesized by GenScript and sub-cloned from pUC57 into pOEM1.

Creation of RL001 by deletion of tolC from *E. coli* GB2005 genome

To create a strain compatible with TolC selection/counterselection (RL001), the genomic copy of TolC was deleted from GB2005 cells by electroporation with the oligo B22I3 followed by selection for resistance to Colicin E1 as described (“TolC Cassette Removal”). The correct deletion was confirmed by PCR using the flanking primers B22E8 and B22F1.

Insertion of TolC cassettes into bacmids via recombineering

TolC cassettes were generated by PCR from plasmid D1495 and targeted for integration into bacmids via 50-bp homology arms. Primer design followed the general scheme -- 5' -N50-TTGAGGCACATTAACGCCCTAT-3' and 5' -N50-CCCCGTCGTCGTCATCAG-3' where ‘N50’ represents the 50 nucleotides homology arms. Transformation of bacmid strains with pSC101-BAD-gba-Cm conferred recombineering proficiency after transient arabinose-mediated induction of the phage recombinase activities. Competent cells, prepared after 45 min of arabinose induction at 37°C, were then electroporated with the tolC cassettes. Following addition of 1ml of LB the cells were shaken for 2 h at 37°C then plated on kanamycin and 0.01% SDS to select for integration of the cassette into the bacmid. Single colonies were then re-streaked to confirm resistance to 0.01% SDS and to demonstrate sensitivity to Colicin E1 (0.1µg/ml). PCR, using flanking primers, was then used to screen for correct integration into the targeted region and this was conﬁrmed by DNA sequencing of purified bacmids.

TolC cassette removal

To remove TolC cassettes from bacmids, strains were transformed with pSC101-BAD-gba-Cm plasmid to allow recombineering proficiency after transient arabinose-mediated induction of phage recombinase activities. Electrocompetent cells were then prepared from these transformants, and electroporated with 500ng of an oligonucleotide with homology to sequences flanking the insertion site of the TolC cassette. After addition of 1ml of LB the cells were grown for 4h at 37°C, then selected for deletions by adding Colicin E1 to 1000 µg/mL and shaking for an additional 1 hr prior to plating on antibiotics (kanamycin, 0.1 µg/mL colicin E1). ColicinE1 resistant transformants were screened for proper removal of tolC by PCR using flanking primers and the loss of tolC was conﬁrmed by streaking single colonies on agar plates containing 0.1µg/ml colicin E1.

Deletion of ORF1629 (AcOrf-9) from bMON14272

*E. coli* strain RL001 was transformed with bMON14272, the bacmid containing the baculovirus genome isolated from *E. coli* DH10Bac cells, and pSC101-BAD-gba-Cm. This strain (H085 strain, Table S2) was electroporated with 300ng of gel purified tolC_cassette1 (Table S1). SDS resistant transformants were screened by PCR for correct insertion of the tolC cassette into ORF1629 region using primers B21I6 and B22F2 to isolate strain H088 (RL001 bMON14272[*∆AcORF1629 tolC*]). Removal of the tolC cassette followed the general strategy described above in "TolC cassette removal", using 0.5µg of oligo B23D1. Colicin E1 resistant transformants were PCR screened for proper removal of tolC with primers B22F2 and B21I5. Glycerol stocks of RL001 bMON14272[∆*AcORF1629*] (H090 strain, Table S2) were prepared.

Deletion of lef2 (AcOrf-6) and ORF603 (AcOrf-7) from bMON14272

H090 cells harboring pSC101-BAD-gba-Cm were electroporated with 160ng of gel purified tolC_cassette2 (Table S1). SDS resistant transformants were PCR screened for proper positioning of tolC in the bMON14272 lef2 region with primers B24C4 and B2C4. Glycerol stocks of RL001 bMON14272[∆1629 ∆lef2 tolC] (H091 strain, Table S2) were prepared. Removal of the tolC cassette used 0.5µg of oligo B24B8. Colicin E1 resistant transformants were screened for proper removal of tolC with primers B24C4 and B24D1. Glycerol stocks of RL001 bMON14272*[∆1629 ∆lef2*] (H092 strain, Table S2) were prepared.

Deletion of chitinase (AcOrf-126) and cathepsin (AcOrf-127) from bMON14272

H092 cells harboring pSC101-BAD-gba-Cm were electroporated with 40ng of gel purified tolC_cassette3 (Table S1). SDS resistant transformants were PCR screened for proper positioning of tolC in the bMON14272 lef2 region with primers B27E3 and B27E4. Glycerol stocks of RL001 bMON14272*[∆AcORF1629 ∆cathepsin ∆chitinase ∆lef2 tolC*] (H097 strain, Table S2) were prepared. Removal of the tolC cassette used 0.5µg of oligo B27H5. Colicin E1 resistant transformants were PCR screened for proper removal of tolC with primers B27E3 and B27E4. Glycerol stocks of RL001 bMON14272[*∆AcORF1629 ∆cathepsin ∆chitinase ∆lef2*] (strain, Table S2) were prepared.

Insertion of Furin in bMON14272

H099 cells harboring pSC101-BAD-gba-Cm were electroporated with 300ng of purified tolC-furin_cassette (Table S1). SDS resistant transformants were PCR screened for proper positioning of tolC-furin in the conotoxin region with primers B30A1 and B30A3. Glycerol stocks of RL001 bMON14272[*∆AcORF1629 ∆cathepsin ∆chitinase ∆lef2 ∆conotoxin tolC-furin*] (H131 strain, Table S2) were prepared. Removal of the tolC cassette used 1.5µg of oligo B30D8. Colicin E1 resistant transformants were PCR screened for proper removal of tolC with primers B30A1 and B30A3. Glycerol stocks of RL001 bMON14272[*∆AcORF1629 ∆cathepsin ∆chitinase ∆lef2 + furin*] (H133 strain, Table S2) were prepared.

Bac-to-bac bMON14272:Activin A bacmid

pFastBacM11Activin A was transformed into *E. coli* DH10Bac cells, resulting in the bMON14272:Activin A recombinant bacmid which was identified by blue/white screening as described in the Bac-to-Bac protocol.

**Supplemental references**

Constam, D.B., and Robertson, E.J. (1999). Regulation of bone morphogenetic protein activity by pro domains and proprotein convertases. J Cell Biol *144*, 139-149.

**Supplemental tables**

| **Table S1** | **TolC cassettes** | |  |
| --- | --- | --- | --- |
|  |  |  |  |
| **Cassette** | **Template** | **Primers** | **Product size (kbp)** |
| tolC_cassette0 | GB2005 | B22A2, B22A3 | 1.6 |
| tolC_cassette1 | D1495 | B22F3, B22F4 | 1.6 |
| tolC_cassette2 | D1495 | B24B9, B24C1 | 1.6 |
| tolC_cassette3 | D1495 | B27H7, B27B5 | 1.6 |
| tolC-furin_cassette | D1678 | B29I4, B30D3 | 4.6 |

| **Table S2** | ***E. coli* strains** |
| --- | --- |
|  |  |
| **Name** | **Genotype** |
| H085 | RL001 bMON14272 pSC101-BAD-gba-Cm |
| H088 | RL001 bMON14272[∆1629 +tolC] |
| H090 | RL001 bMON14272[∆1629] |
| H091 | RL001 bMON14272[∆1629 ∆lef2 +tolC] |
| H092 | RL001 bMON14272[∆1629 ∆lef2] |
| H097 | RL001 bMON14272[∆1629 ∆cathepsin ∆chitinase ∆lef2 +tolC] |
| H099, DefBac | RL001 bMON14272[∆1629 ∆cathepsin ∆chitinase ∆lef2] |
| H131 | RL001 bMON14272[∆1629 ∆cathepsin ∆chitinase ∆lef2 ∆conotoxin +tolCFurin] |
| H133, DefBac^Fur+^ | RL001 bMON14272[∆1629 ∆cathepsin ∆chitinase ∆lef2 ∆conotoxin, +Furin] |
